# Recurrence of an Osteo-Cartilaginous Tumor, Connected with the Nasal Process of the Superior Maxilla; Second Removal; Recovery

**Published:** 1852-10

**Authors:** 


### ARTICLE XXII.

*Recurrence of an Osteo-cartilaginous Tumor, connected with the Nasal Process of the Superior Maxilla; Second Removal; Recovery.* Under the care of Mr. Partridge.

Amongst the tumors which are apt to re-appear on the cicatrix, or near it, after removal, is the cartilaginous tumor; and though the recurrence of the growth may perhaps not be very alarming, it is nevertheless a matter of great importance to the patient, as a malignant degeneration has, in similar cases, been known to occur. One of the latest authorities on innocent and malignant tumors expresses himself as follows:—"We must conclude, I think, from these cases, that although the general rule of innocence of cartilaginous tumors is established by their usual history, by numerous instances of permanent health after removal, and by cases in which, after death, no similar growths are found in lymphatics or internal organs, yet recurrence after operation may ensue. And I think that when this happens, it will generally be found that the recurring growths, if not the original growths also, are soft, rapid in their increase, and apt to protrude and destroy adjacent parts, (see below the destruction of turbinated bones;) as if we had again, in these, an instance of that gradual approximation to the completely malignant characters, of which I spoke in the last lecture." (Paget—"Lectures on Tumors, delivered at the Royal College of Surgeons, in May, 1851," page 70.)

The following case, taken from the notes of Mr. Ulermark, dresser of the patient, offers an instructive example of such a recurrence.

Henry M——, aged nineteen, a potboy, was admitted, April 24, 1852, under the care of Mr. Partridge. Twenty months since, the patient perceived a small pimple on the right side, about half way down the nose, which increased slowly in size for eight months, when he was admitted into St. Thomas'

Hospital, under the care of Mr. Le Gros Clark. The latter removed the tumor, which was then the size of a small walnut, very hard, and probably fibrous; the wound healed up in a month, and the boy was discharged. Three months before his admission into King's College Hospital, he caught cold, and the upper lip of the right eye became swollen. The swelling soon changed to the site of the old tumor, and has continued increasing up to the present time; it is now as large as a hazelnut, and is distinctly fluctuating.

The second day after admission, the abscess burst, and discharged a good deal of purulent matter; twenty-three days afterwards, the part presented the following characters:—The tumor is about the size of a large walnut, oblong in shape, and seems to be immediately under the skin; it does not cause any projection into the mouth, nor is the eyelid at all elevated by it, and there is no evident enlargement of the anterior wall of the antrum. The patient can breathe well through the nostril, though a superficial abscess has again burst.

On June 5, 1852, forty-one days after the boy came into the hospital, Mr. Partridge proceeded to remove the tumor. He began by making an incision from the inner angle of the orbit, by the side of the nose, along the base of the tumor, cutting through the upper lip a little to the right side; and the integuments being turned back, the external surface of the tumor was completely exposed. The latter was evidently of an osteo-cartilaginous character, and projected slightly into the antrum, involving the nasal process of the superior maxillary bone. The tumor was easily removed, as were also both the superior and inferior turbinated bones, on account of their hanging loose in the cavity of the nose. The septum and roof of the nasal fossa were quite sound. The lip was secured by long pins, and five sutures put on the margin of the wound.

Sixth day after the operation.—On the removal of the pins, the parts were found to have united, except at the internal angle of the mouth. The nose was ordered to be syringed with lukewarm water. A good deal of matter was for a few days discharged from the posterior nares into the mouth, but the patient went on extremely well, and was made an out-patient June 25, twenty days after the operation, the whole wound being healed, except by the inner canthus of the eye.—*London Lancet.*

### BIBLIOGRAPHICAL.

